# Pet’s influence on humans’ daily physical activity and mental health: a meta-analysis

**DOI:** 10.3389/fpubh.2023.1196199

**Published:** 2023-05-30

**Authors:** Catarina F. Martins, Jorge P. Soares, António Cortinhas, Luís Silva, Luís Cardoso, Maria A. Pires, Maria P. Mota

**Affiliations:** ^1^Research Centre in Sports Sciences, Health and Human Development (CIDESD), Department of Sport, Exercise and Health Sciences, School of Life and Environmental Sciences (ECVA), University of Trás-os-Montes and Alto Douro (UTAD), Vila Real, Portugal; ^2^Animal and Veterinary Research Centre (CECAV), Department of Veterinary Sciences, School of Agrarian and Veterinary Sciences, University of Trás-os-Montes and Alto Douro (UTAD), Vila Real, Portugal; ^3^Associate Laboratory for Animal and Veterinary Sciences, University of Trás-os-Montes and Alto Douro (UTAD), Vila Real, Portugal

**Keywords:** active lifestyle, health, pet ownership, physical activity, quality of life

## Abstract

**Abstract:**

The benefits of the human-animal bond on owners’ health and quality of life have been the focus of research in recent decades. However, the results are still inconsistent. Thus, this study aims to investigate whether the presence of a pet, compared to a control group, influences daily physical activity levels and mental health using a meta-analytic method.

**Methods:**

The PubMed, Web of Science, and Scopus databases were searched for all research articles that included pets as an object of study and related mental health and quality of life variables between pet owners and non-owners until April 2022. The PRISMA 2020 checklist was used, and the Downs and Back checklist was used to assess the methodological quality of the studies. Standardized mean differences and 95% confidence intervals were used to assess the difference between a group of pet owners and non-pet owners.

**Results:**

An initial search located 11,389 studies, but only 49 studies fulfilled all requirements. Our results indicate that pets have a moderately significant positive effect on the physical activity of owners compared to non-pet owners. Among the moderating variables, the frequency of physical activity showed a highly significant effect, indicating that owners had a higher frequency of physical activity than non-owners. Moreover, our results indicate a significant impact but with a low effect size of pets on owners’ mental health when compared to non-pet owners.

**Conclusion:**

Pet ownership does not seem to influence owners’ mental health, but it does influence their physical activity. Specifically, owners show a higher frequency of physical activity than non-owners.

## 1. Introduction

Pets play an important role in human life and human health (1). Improvements in physical, mental, psychological, and social health have been described in several works (1–7).

Physical activity (PA) is a determinant of health and quality of life and has been indicated for the prevention and treatment of various diseases (8, 9). There is also evidence that the strength of the relationship between owner and pet is strongly associated with increased PA (10, 11). Once again, dogs are the most commonly reported animals related to the increase in PA (7, 12) possibly due to social support (13), increased motivation to exercise (14, 15), or even the sense of responsibility to take care of the pet (16). Responsibility is often highlighted as a potential strategy to increase PA levels in older adult individuals (10, 17, 18) and in general population (19). However, confounding variables such as housing conditions, pet attachment, and the number of household members can modify the frequency of walks with the pet and interfere with the magnitude of the results.

Nowadays, mental health is one of the main global concerns, with an estimated 970 million people in the world having a mental disorder (20). A mental disorder is a syndrome characterized by cognitive, emotional, or behavioral dysfunction that reflects an impairment in the psychological, biological, or developmental processes underlying mental and behavioral functioning (20). These disorders not only have an impact on an individual’s daily life but also entail substantial costs to society (21). According to the OECD, up to 13% of total health spending is directed toward mental health services (22). According to Statista Research, Portugal invested approximately 136.2 million Euros in mental health hospitals in 2019 (23). As a result, several studies have investigated the possible influence of pets on human mental health, including loneliness, depression, anxiety, stress, satisfaction with life, happiness, social support, and other factors.

Interactions with pets have positive influences on the owner, with overall positive effects on mental health, such as reducing depression and anxiety (6, 24). Moreover, owning a pet may increase social connections (25–27). The human-animal bond, strengthened by the acquisition of a pet, is associated with psychological and physical benefits in children, adults, and elders (6, 10, 25, 28, 29). These aspects have a significant overall effect on the mental health of the human population, as their continued failure or dysfunction can translate into poor mental health, possibly contributing to increased morbidity and mortality (30–32). Growing evidence indicates that pets may trigger feelings of comfort, security, and emotional support, which probably have positive effects on humans by counteracting feelings of anger, sadness, anxiety, and depression (24, 33, 34). Considering the importance of social health, evidence supports that relationships with pets confer similar support to humans (35, 36), particularly in cases of mental disorders (37). Dogs have been proposed as promoters for the initiation of shared interpersonal interactions that enhance social networks (e.g., daily walks) (38). Despite the growing literature, contradictory results have been described regarding different human dimensions, namely human health variables and quality of life (15, 39). A possible explanation may rely on the value that the family or the subject gives to the pet, which may interfere with the overall mental and physical benefits of the pet’s relationship (40). Some authors have suggested a negative influence of the pet’s non-psychological parameters (41, 42) such as lower psychological well-being (18, 30), depression (41), and anxiety (41) compared to Non-Pet Owners (NPOs). Moreover, pet owners (POs) showed a lower perception of health as well as a higher prevalence of disease than non-pet owners (NPOs) of different ages, which may contribute to a worse quality of life in specific situations such as the COVID-19 pandemic (43). Although some studies have pointed out this trend, Mueller et al. (4) highlighted that POs may have adopted the pet as a way to cope with depressive symptoms or other mental disorders they were already experiencing.

As mentioned, despite a large number of studies, some results are contradictory, possibly due to the different variables considered in each study and the different study designs. To our knowledge, no meta-analysis considering this evidence has been published. Therefore, this article aims to (a) estimate the levels of physical activity (PA) of pet owners (PO) and non-pet owners (NPO) and their relation with the quality of life and human health, and (b) quantify the effect of pets on mental health and, consequently, on the quality of life of human beings. The hypotheses tested in this meta-analysis are: H1: PO tends to show higher levels of daily PA than NPO. H2: Pets have a significant and positive influence on the mental health of PO. H3: PO shows better results regarding anxiety, loneliness, depression, stress, life satisfaction and happiness, social support, quality of life, health and well-being, general mental health and resilience, and mood and self-regulation (affections, emotions, relationships) than NPO.

## 2. Methods

### 2.1. Search strategy

Electronic database searches were conducted in PubMed, Web of Science, and Scopus for all articles published before April 2022, following the Preferred Reporting Items for Systematic Reviews and Meta-Analyses (PRISMA) guidelines (44, 45). The search terms were “Pets” OR “Pet” OR “Pet companion” OR “Pet owner” OR “Human-animal relationship” OR “Pet-interaction” OR “Dog walking” AND “Human health” OR “Quality of life” OR “Benefit” OR “Mental health” OR “Physical health” OR “Health” OR “Life satisfaction” OR “Well-being.” The search was adapted for each database as needed, and filters were used to exclude observational studies, reviews, posters, and other studies that were not eligible for meta-analysis.

### 2.2. Inclusion/exclusion criteria

No studies were excluded based on the type of pet selected since the focus of this study is to investigate the influence of pet ownership on human mental health, daily physical activity, and quality of life. The inclusion criteria for article selection include reporting the impact of animals on human mental health or quality of life, having a control group without any kind of pet, statistical treatment and feasible data for meta-analysis, and writing in English, Portuguese, or Spanish. Articles are excluded if they are reviewing articles, systematic reviews, meta-analyses, or conference reports, have no focus on live animals (for example, robot pets), use pets for animal-assisted therapy, or are studies conducted with working animals. Study selection parameters were not limited to the year of publication, intra-human variability, age of participants, or sample size since the goal of this meta-analysis was to conduct a comprehensive search. However, studies that were published only in abstract form or were not accessible via inter-library loan were excluded from this meta-analysis. Study eligibility was determined individually by the group members, with each study classified as include, exclude, or unclear. Articles that were classified as “include” or “unclear” by both reviewers were included for full-text review, and any discrepancies in the determination of study eligibility were resolved through mutual consensus.

### 2.3. Review strategy

After the articles were searched, duplicates were removed using Zotero. The article selection process involved screening the titles and abstracts for inclusion and exclusion criteria. In cases of doubt, the articles were read in their entirety to verify if the study design was suitable for the aim of this meta-analysis. Another reviewer then checked all the excluded and included articles for validity. Subsequently, the included articles were read in their entirety by two reviewers, and any inclusion/exclusion conflicts were resolved by a third and fourth reviewer. An acceptable concordance rate of 90% was predefined. The reviewers achieved a concordance rate of 93%, resolving 19 inclusion/exclusion conflicts.

The references of the included articles were manually searched to identify possible relevant articles that were not included in the initial search, to achieve a wider scope of relevant studies and reduce publication bias. This search was conducted from December 20th, 2021 to April 3rd, 2022.

### 2.4. Data extraction

Data from each study included in the meta-analysis were double-extracted by two authors into a table using Microsoft Excel software. Disagreement between the extractors, which consisted mainly of small additional details, was easily resolved between all authors.

Data extracted from each study included: title, author(s), journal, year of publication, study, characteristics (date of data collection, study design), participant characteristics (age, sample sizes), outcomes, intervention description, control condition description, adverse effects, adherence, dropouts, and results.

The identified studies were divided into two groups and assigned to two pairs of reviewers, who independently conducted data extractions and assessed the quality of the studies using the Downs and Black (DB) quality assessment tool (46). The DB tool consists of 27 criteria that assess study reporting (10 items), external validity (3 items), and internal validity, including design, bias, and power (14 items). The maximum score achievable is 27 points. For cross-sectional studies, the modified version of the DB tool was used, which includes 16 criteria that assess study reporting (9 items), external validity (2 items), and internal validity, including design, bias, and power (5 items). The maximum achievable score was 16 points. Any discrepancies in the DB scoring were resolved through consensus among the reviewers (Supplementary material A).

### 2.5. Data analysis

The Comprehensive Meta-Analysis V2.2.057 software was used for the meta-analysis. Design-specific meta-analyses were conducted for cohort and cross-sectional studies on mental health and PA. Preference was given to the use of mean and standard deviation, and if the not possible, mean difference with a 95% CI was used in all analyses. Separate analyses were performed for PA and mental health.

The statistical heterogeneity was assessed using the Cochran Q (47) test and I^2^ statistics (48). We used the random effects model and set the significance level at *p* ≤ 0.05. We evaluated the risk of publication bias by visually inspecting the funnel plot and using the Egger test (49) and Begg’s test (50). Subgroup analyses of mental health were performed by grouping the age of the sample into three categories: children and adolescents (≤18), adults (≥18 to ≤60), and older adults (≥60). Subgroup analyses also included domain-specific analyses of mental health, such as loneliness, depression, anxiety, stress, life satisfaction and happiness, social support, quality of life, health and well-being, general mental health and resilience, and humor and self-regulation (affections, emotions, relationships). Subgroup analyses of PA considered the domain-specific of physical activity (measured by minutes, frequency, counts, and mets), as well as the form of data collection and defined age groupings. Notably, if a study reported results for more than one specific type or domain of PA separately (e.g., walking and gardening), all types of PA were included in the analysis as independent variables. However, if a study also reported on the wider spectrum of physical activity measures (e.g., total PA, total LTPA), only the broader measure was used to avoid duplication.

## 3. Results

### 3.1. Search and screening

**Figure 1:**
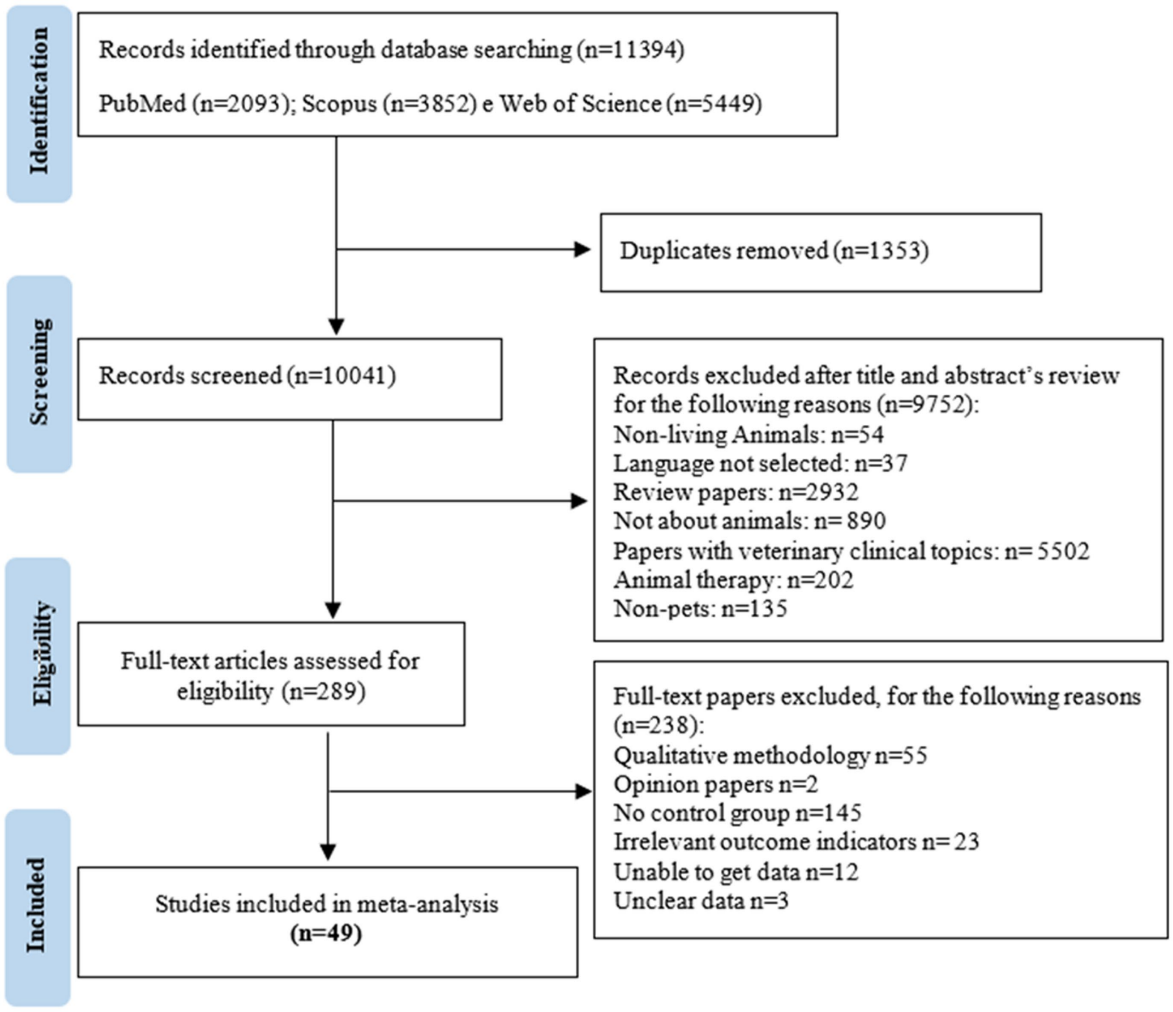
Flowchart of the search strategy to include studies.

A total of 11,389 records were identified in the electronic databases. After removing duplicates and articles irrelevant to the analysis, 289 full-text publications were assessed for eligibility. Based on the inclusion and exclusion criteria, 238 articles were also excluded. A total of 49 articles met the eligibility criteria and were included in this meta-analysis. Details of the search strategy are provided in Figure 1.

### 3.2. Included study characteristics

From a total of 320,971 participants, 10,233 were children and adolescents, 79,108 adults, and 2,308 were old adults.

Of the 49 included studies that evaluated pet ownership and its influence on mental health and daily PA parameters. Regarding mental health, 27 were concerned (4, 24, 25, 30, 43, 51–73). The distribution of these articles by subcategory of mental health can be observed in Table 1. Relative to physical activity 22 studies were considered for analysis (10, 12, 13, 16, 29, 74–77, 79–90). Additionally, 5 studies were included in both analyses (13, 74–77). Descriptive data of the included studies are presented in Tables 2, 3 for PA and mental health, respectively.

**Table 1:** Distribution of articles by subcategory of mental health.

1	2	3	4	5	6	7	8	9	Author^a^
									Amiot et al. (30)
									Antonacopoulos (51)
									Ballin et al. (74)
									Black (25)
									Bennett et al. (24)
									Bradley and Bennett (52)
									Branson et al. (53)
									Brkljacic et al. (54)
									Canady and Sansone (55)
									Carr et al. (75)
									Cloutier and Peetz (56)
									Curl et al. (57)
									Endo et al. (58)
									Feng et al. (76)
									Grajfoner et al. (59)
									Hajek and König (60)
									Hill et al. (61)
									Kim and Chun (62)
									Mičková et al. (77)
									Mueller et al. (78)
									Muldoon et al. (63)
									Muraco et al. (64)
									Phillipou et al. (43)
									Pruchno et al. (65)
									Ramírez and Hernández (66)
									Reis et al. (67)
									Roux and Wright (68)
									Taniguchi et al. (13)
									Teo and Thomas (70)
									Watson and Weinstein (71)
									Wright et al. (72)
									Wright et al. (73)

1 – Loneliness; 2 – Depression; 3 – Anxiety; 4 – Stress; 5 – Life satisfaction and happiness; 6- Social Support; 7 – Quality of life, health and well-being; 8 – General mental health and resilience; 9 – Humor and self-regulation (affections, emotions, relationships).

a The articles have been arranged in alphabetical order.

**Table 2:** Studies reference to physical activity.

Physical activity (PA)											
*The information in columns’ purpose and major findings are quoted directly from the original publications.											
						Collected data					

Author (year)^a^	Country	n	Population	Pet	Purpose	Intervention methods	Evaluation exercise	Pet owner M(SD) or IC	Non-owner M (SD) or IC	Major findings	Quality score
Ballin (74)	Sweden	DO = 199; NDO = 1,207	Older adult	Dog	“Investigated the association of DO with accelerometer-measured PA of different intensities and daily steps in 70-year-old individuals”	Accelerometer: LPA Accelerometer: MVPA Accelerometer: Steps	Mins/wk Mins/wk Counts	283.2 (77.4) 43.4 (30.3) 8,712 (3724)	266.7 (75.8) 32.6 (23.1) 7,131 (2932)	“DO was associated with higher levels of daily LPA. MVPA. and steps”	15
Byers (80)	USA	DO = 10; NDO = 21	Adults	Dog	“Understanding how owner-pet bonding can leverage increased PA for owner and pet”	Pedometer: Steps	Counts/day	1st Evaluation 8,040 (978) 2nd Evaluation 8,734 (3252)	1st Evaluation 8,349 (972) 2nd Evaluation 8,940 (2845)	“Both groups increased the number of daily steps from pre to post”	24*
Brown and Jensen (79)	USA	T = 536	Adults	Dog	“Examines whether perceived and audited walkability and activity differentiate across three dog owner and walker groups. With separate analyses across 2 years”	IPAQ: Walk to get places IPAQ: Walk for leisure Accelerometer: LPA Accelerometer: MVPA	Mins/wk Mins/wk Mins/day Mins/day	1st Evaluation 326.20 (422.06) 2nd Evaluation 281.24 (382.86) 1st Evaluation 289.83 (386.34) 2nd Evaluation 383.48 (445.86) 1st Evaluation 216.76 (52.45) 2nd Evaluation 221.41 (53.34) 1st Evaluation 19.97 (17.05) 2nd Evaluation 20.70 (17.70)	1st Evaluation 311.27 (374.55) 2nd Evaluation 300.08 (389.98) 1st Evaluation 233.27 (339.61) 2nd Evaluation 269.74 (370.29) 1st Evaluation 210.21 (60.90) 2nd Evaluation 215.57 (64.78) 1st Evaluation 20.59 (18.08) 2nd Evaluation 21.75 (18.31)	“Dog walkers reported high levels of leisure walking. But these high levels were not corroborated by objective accelerometer measures”	23*
Brown and Rhodes (16)	Canada	DO = 70; NDO = 281	Older adults	Dog	“Examined the relationship between walking. PA levels. and potential psychological mediators between people who owned dogs and those who did not own dogs in the Capital Region District of Greater Victoria. British Columbia. Canada”	GLTEQ: Mild walking GLTEQ: Moderate walking GLTEQ: Strenuous walking GLTEQ- Walking GLTEQ- Mild PA GLTEQ- MPA GLTEQ- Strenuous PA	Freq/wk Mins/wk Mins/wk Mins/wk Freq/wk Mins/wk Mins/wk	137.79 (182.72) 136.39 (164.46) 28.43 (64.19) 300.18 (223.38) 155.62 (189.30) 164.93 (179.79) 82.71 (108.05)	59.27 (89.43) 89.55 (111.54) 19.80 (60.79) 168.38 (163.62) 96.41 (131.37) 115.21 (129.54) 72.30 (127.19)	“The analyses revealed that dog owners spent more time in mild and MPA and walked an average of 300 min per week compared to non– dog owners who walked an average of 168 min per week”	15
Carr (75)	Canada	DO = 20; NDO = 36	Adults with chronic low back pain	Dog	“Explore whether a relationship exists between dog ownership and well-being for people with chronic LBP”	GLTEQ	Mins/wk	56.95 (11.23)	56.81 (15.42)	“The two groups do not differ significantly in their physical functioning or physical health”	13
Coleman (81)	USA	DO walker = 429; DO non-walker = 183; NDO = 1,578	Adults	Dog	“Examined how demographics PA. weight status. and neighborhood characteristics varied among households with and without dogs”	Accelerometer: MVPA	Mins/day	Owner walker 35 (24) Non-walker 27 (21)	33 (24) 33 (24)	“Dog walking was associated with a higher proportion of participants who met national recommendations for MVPA when compared to non-dog owners”	15
Corrigan (82)	USA	DO = 54; DO = 57 DO = 74; NDO = 28; NDO = 32; NDO = 33	Adults	Dog	“Determine whether there was a relationship between dog ownership and PA”	IPAC: MPA IPAC: VPA IPAC: Walking	Mins/wk Mins/wk Mins/wk	87.81 (136.23) 116.56 (116.84) 223.53 (188.36)	34.69 (41.62) 80.73 (84.1) 115.27 (119.2)	“Dog ownership was significantly associated with meeting physical activity guidelines in veterinary students”	16
Curl (17)	USA	DO = 173; NDO = 500	Older Adults	Dog	“Explored the associations between dog ownership and pet bonding with walking behavior and health outcomes in older adults”	Self-reports: Frequency MPA Self-reports: Frequency VPA	Freq/wk Freq/wk	Owner walker 2.48 (0.09) Non-walker 1.76 (0.16) Owner walker 1.69 (0.12) Non-walker 0.95 (0.16)	2.10 (0.07) 2.10 (0.07) 1.09 (0.08) 1.09 (0.08)	“Dog walking was associated with more frequent moderate and vigorous exercise and was associated with better physical health or health behaviors”	16
Dall (10)	United Kingdom	DO = 46; NDO = 42	Older adults	Dog	“Measures of PA and sedentary behavior (SB) provide opportunities to gain insight into both the intensity and pattern of PA and SB. allowing closer scrutiny of the potential relationship between dog ownership and health”	Accelerometer: Walking Accelerometer: Walking at a moderate cadence Accelerometer: Standing Accelerometer: Sedentary	Mins/day Mins/day Hours/day Hours/day	119 (109. 131) 32 (23. 43) 4.44 (4.13. 4.75) 9.94 (9.54. 10.35)	96 (88. 106) 11 (8. 15) 4.35 (4.04. 4.66) 10.25 (9.84. 10.66)	“Owning a dog indicated a large. Potentially health improving. The average effect of 22 min additional time spent walking and 2,760 additional steps per day. With this additional walking undertaken at a moderate intensity cadence. Dog owners had significantly fewer sitting events”	14
Feng (76)	United Kingdom	DO = 50; NDO = 497	Older adults	Dog	“Examine whether dog ownership amongst community dwelling older adults is associated with objectively measured PA”	Accelerometer	Counts	180.853 (13.257)	142.71 (3469)	“Dog ownership is associated with PA in later life”	16
Dall (83)	USA	DO = 36,984; NDO = 115,645	Postmenopausal women	Dog	“Examine cross-sectional associations between dog ownership and PA measures in a well-characterized. Diverse sample of postmenopausal women”	WHIPAQ: Walking WHIPAQ: Total WHIPAQ: Walking WHIPAQ: Total	Mins/wk Mins/wk MET hour/wk MET hour/wk	87.5 (100.7) 176.8 (182.7) 4.60 (5.92) 11.9 (13.8)	87.2 (99.9) 182.8 (178.9) 4.71 (6.03) 12.6 (13.7)	“Dog ownership is associated with increased PA in older women. Particularly among women living alone. Health promotion efforts aimed at older adults should highlight the benefits of regular dog walking for both dog owners and non-dog owners”	16
Koohsari (84)	Japan	DO = 119; NDO = 574	Adults	Dog	“Examined the associations between dog ownership with objectively-assessed sedentary behaviour and PA among a sample of middle-aged adults in Japan”	Accelerometer: Total sedentary time Accelerometer: sedentary bouts Accelerometer: LPA Accelerometer:MPA Accelerometer:VPA Accelerometer: MVPA	Mins/day Mins/day Mins/day Mins/day Mins/day Mins/day	473.1 (129.9) 155.7 (99.0) 376.9 (115.6) 74.0 (40.1) 1.9 (8.8) 69.2 (38.7)	506.3 (117.6) 175.9 (91) 344.7 (109.1) 67.3 (37.4) 1.9 (5.6) 75.9 (41.7)	“Owning a dog is associated with several types of adult sedentary behaviors. But not medium to high intensity PA”	15
Lail (85)	Canada	DO = 115; NDO = 313	Adults	Dog	“Investigated the extent to which dog-ownership influences seasonal patterns in neighbourhood-based walking among adults living in highly-variable climate”	NPAQ: Walking for recreation (summer) NPAQ: Walking for recreation (winter) NPAQ: Walking for transportation (summer) NPAQ: Walking for transportation (winter)	Mins/wk Mins/wk Mins/wk Mins/wk	213.6 (206.8) 253.2 (211.8) 59.1 (128.2) 59.9 (112.6)	123.3 (157.7) 107.1 (135.9) 74.9 (123.7) 69.8 (119.3)	“Dog-owners reported more walking for recreation in their neighbourhoods than did non-owners. Both in summer and in winter. Dog-owners and non-owners did not differ in the amount of walking that they reported for transportation. Either in summer or in winter”	16
Machová (86)	Czech Republic	PO = 60; NPO = 51	young adult women	Any type of pet	“Compare PA levels between animal owners and non-owners and to research potential differences between owners of different kinds of animals”	IPAQ: VPA IPAQ: VPA IPAQ: MPA IPAQ: MPA IPAQ: WPA IPAQ: WPA IPAQ: Total PA IPAQ: Total PA	Mins/wk MET-min/wk Mins/wk MET-min/wk Mins/wk MET-min/wk Mins/wk MET-min/wk	77.5 (105) 1920 (3840) 60 (150) 900 (2280) 120 (120) 2,772 (2772) 294 (240) 6212 (4772)	60 (70) 1,080 (2400) 60 (78) 320 (960) 120 (120) 2,376 (2772) 210 (180) 3,990 (3363)	“Animal owners generally reported higher PA levels compared to people who do not own any pets”	13
Michová (77)	Czech Republic	DO = 26; NDO = 18	Older adults	Dog	“To see if dog ownership affects PA. sleep and self-reported health in a group of older adult people”	Accelerometer: Activity time Accelerometer: Steps IPAQ: VPA IPAQ: MPA IPAQ: Walking IPAQ: VPA IPAQ: MPA IPAQ: Walking IPAQ: Sitting	Mins/day counts/day Mins/wk Mins/wk Mins/wk MET-Min/wk MET-Min/wk MET-Min/wk Mins/day	127 (62) 9,961 (5213) 50 (70) 73 (52) 128 (48) 1,123 (1847) 700 (589) 2,910 (1114) 353 (125)	73 (28) 5,247 (2644) 8 (29) 52 (49) 99 (58) 173 (678) 447 (619) 1904 (1143) 363 (142)	“Dog-owners reported higher total PA time (min/week). MET/min/week spent in walking. and spent calories/week than non-owners”	14
Oka and Shibata (87)	Japan	DO = 930; PO = 793; NPO = 1733	Adults	Any type of pet	“Examined the association between dog ownership and health-related PA among Japanese adults”	IPAC- MVPA IPAC- Walking IPAC- Sedentary behaviour	MET hour/wk MET hour/wk MET hour/wk	DO 17.0 (1.159) Any pet 10.9 (1.229) DO 12.4 (0.757) Any pet 10.5 (0.802) DO 6.4 (0.135) Any pet 6.9 (0.143)	11.7 (0.593) 11.7 (0.593) 9.8 (0.387) 9.8 (0.387) 6.9 (0.069) 6.9 (0.069)	“The dog owners had higher physical activity levels than owners of other kinds of pets and those without any pets. Suggesting that dogs may play a major role in promoting PA”	14
Richards (12)	USA	DO walker =1,012; DO non-walker =221; NDO = 2,262	Adults	Dog	“The purpose of this study is to determine whether dog owners who walk their dog participate in more PA than dog owners who do not walk their dog and non–dog owners”	Self-reports: MVPA	Mins/wk	Owner walker 200.5 (14.8) Non-walker 198.0 (13.1)	178.3 (11.0)	“Most dog owners did not walk their dog. Dog owners were not more active than non–dog owners. Except when considering the activity obtained via dog walking”	16
Schofield (88)	Australia	DO = 646; NDO = 591	Adults	Dog	“To understand whether dog owners were actually involved in walking their dog. and their feelings about the usefulness of dog ownership for PA”	TAAQ: Accumulated PA TAAQ: Walking for leisure	Mins/wk Mins/wk	334.8 (408.6) 114.9 (197.9)	346.4 (414.9) 108.2 (178.8)	“Results showed that the simple presence of a household dog displayed no relationship to the acquisition of sufficient levels of PA in the overall population”	14
Taniguchi (13)	Japan	PO = 1,545; NPO = 6,377	Older adults	Dog Cat	“Examined physical function. PA. social function. and psychological function of a population of community-dwelling older Japanese dog and cat owners after controlling for important confounders”	IPAC: VPA IPAC: MPA IPAC: Walking IPAC- MVPA	MET hour/wk MET hour/wk MET hour/wk MET hour/wk	14.1 (32.1) 8.5 (18.5) 25.4 (24.6) 44.7 (54.8)	14.7 (33.3) 7.9 (19.9) 23.1 (22.9) 43.2 (54.8)	“Analysis of variables related to physical function and PA showed that motor fitness scale and walking activity were significantly associated with experience of dog ownership. After adjustment for important socio-demographic and health characteristics”	16
Thorpe (29)	USA	PO = 594; DO = 96; NDO = 198; NPO = 1939	Older adults	Any type of pet	“Understanding the relationship between pet ownership and PA”	Self-reports: Total time walking Self-reports: Frequency of non-exercise-related walking Self-reports: Frequency of exercise walking Self-reports: Frequency of non-exercise-related walking	Mins/wk Mins/wk Freq/wk Freq/wk	Any pet 69.52 (135.9) DO 75.4 (141.5) NDO 57.8 (123.6) Any pet 2.0 (4.3) DO 2.5 (5.0) NDO 1.1 (2.2) Any pet 1.9 (3.1) DO 2.0 (3.2) NDO 1.6 (2.9) Any pet 2.0 (4.3) DO 2.5 (5.0) NDO 1.1 (2.2)	61.8 (122.3) 1.2 (2.7) 1.7 (2.4) 1.2 (2.7)	“Dog owners were more likely to engage in non-exercise related walking than were non–pet owners. Dog owners reported a greater frequency and duration of walks than either non–pet or non-dog-pet owners. Most of whom had cats”	12
Westgarth (89)	United Kingdom	DO = 166; DO = 184; NDO = 445; NDO = 18 DO = 168; DO = 186; NDO = 444; NDO = 18 DO = 162; DO = 179 NDO = 441; NDO = 17 DO = 165; DO = 183; NDO = 448; NDO = 18 DO = 169; DO = 187; NDO = 449; NDO = 18 DO = 169; DO = 187; NDO = 449; NDO = 18 DO = 169; DO = 187; NDO = 449; NDO = 18 DO = 169; DO = 187; NDO = 449; NDO = 18 DO = 17; NDO = 11 DO = 10; NDO = 36	Adults and Children	Dog	“First aim of this study was to compare the physical activity of dog owners from UK population with people that do not own a dog. A secondary aim of the study was to investigate whether DO spend more or less time than NDO in more intensive PA than walking”	NPAQ: Walk for recreation NPAQ: Walk for transport NPAQ: MVPA NPAQ: VPA NPAQ: Walk for recreation NPAQ: Walk for transport NPAQ: MVPA NPAQ: VPA Accelerometer: Steps Accelerometer: LMVPA Accelerometer: MVPA CAPANS: Walk for recreation CAPANS: Walk for recreation CAPANS: Walk for transport CAPANS: Walk for transport CAPANS: Total PA	Mins/wk Mins/wk Mins/wk Mins/wk Freq/wk Freq/wk Freq/wk Freq/wk Counts Mins/day Mins/day Freq/wk Mins/wk Freq/wk Mins/wk Mins/wk	^b^Dog walker 322.3 (301.7) DO 93 (300) Dog walker 56.8 (117.7) DO 53 (113) Dog walker 131.4 (184.3) DO 126 (180) Dog walker 51.0 (120.5) DO 51,119) Dog walker 7.9 (5.6) DO 7.3 (6.0) Dog walker 2.5 (4.6) DO 2.4 (4.5) Dog walker 3.0 (5.3) DO 2.9 (5.1) Dog walker 0.9 (1.7) DO 0.9 (1.7) Dog walker 7,523 (2710) 297.1 (70.2) 37.8 (20.3) 6.1 (6.4) 115.0 (97.9) 4.0 (4.2) 179.0 (306.9) 1035.0 (1010.0)	NDO 84 (136) NDO Walkers 7.8 (65.5) NDO 75 (123) NDO Walkers 15.8 (42.6) NDO 127 (190) NDO Walkers 80.2 (124.9) NDO 37.1 (91.4) NDO Walkers 52.2 (103.0) NDO 1.6 (2.2) NO Walkers 10.7 (1.99 NDO 3.0 (3.7) NO Walkers 15.8 (42.6) NDO 2.2 (2.9) NDO Walkers 2.0 (2.6) NDO 0.7 (1.5) NDO Walkers 0.9 (1.6) NDO 6,381 (3215) 276.1 (97.6) 30.3 (21.4) 3.4 (6.1) 61.8 (77.2) 6.4 (5.9) 143.1 (127.8) 565.6 (369.2)	“DO were far more likely than NDO to report walking for recreation. and amongst recreational walkers walked for longer per week. Other PA undertaken did not differ by dog ownership. The odds of DO meeting current physical activity guidelines of 150 min per week were four times greater than for NDO. Children with dogs reported more minutes of walking and free-time (unstructured) activity. Dog ownership is associated with more recreational walking and considerably greater odds of meeting PA guidelines. Policies regarding public spaces and housing should support dog ownership due to PA benefits”	15
Yabroff (90)	USA	DO = 7,348; POcat = 5,397; POcat+dog = 3,529; NPO = 25,240	Adults	Dog Cat	“Explored associations between pet ownership and PA in a large. Ethnically diverse population-based sample in California”	Self-reports: Leisure walking Self-reports: Transportation	Mins/wk Mins/wk	Dog 86.1 (1.6) Cat 61.5 (1.8) Dog; Cat 75.7 (2.3) Dog 43.2 (2.4) Cat 46.5 (4.6) Dog; Cat 46.3 (3.7)	64.6 (1.1) 55.1 (1.3)	“Dog owners were slightly less likely to walk for transportation than were non–pet owners but more likely to walk for leisure than non–pet owners in multivariate analyses”	14

NDO, non-dog owner; DO, dog owner; Intervention methods: AAQ, active Australia questionnaire; GLTEQ, Godin leisure time exercise questionnaire; MPA, moderate physical activity; MVPA, moderate a vigorous physical activity; NPAQ, neighborhood physical activity questionnaire; PA, physical activity; VPA, vigorous physical activity; Wk, week; WHIPAQ, women’s health initiative physical activity questionnaire.

* Down’s and Black tool uses 27 criteria.

a Articles arranged in alphabetical order.

b Columns show only the reference values.

**Table 3:** Studies concerning mental health.

Mental Health										
*The information in columns’ purpose and major findings are quoted directly from the original publications.										
							Collected data			
Author (year)^a^	Country	N	Population	Pet	Purpose	Intervention methods	Pet owner M(SD)	Non-pet owner M(SD)	Major findings	Quality score
Amiot (30)	Canada	T = 1,220	Adults	Any type of pet	“Investigate the differences that may exist between pet vs. non-pet owners in terms of their well-being during the COVID-19 pandemic”	UCLA LSS PStressS Vitality PLF COVID (impact)	2.29 (0.55) 4.35 (1.45) 2.79 (0.62) 4.22 (1.33) 4.57 (1.36) 3.60 (1.32)	2.23 (0.52) 4.53 (1.38) 2.74 (0.57) 4.34 (1.28) 4.75 (1.25) 3.44 (1.23)	“Pet owners reported lower well-being than non-pet owners on a majority of well-being indicators; this general pet ownership effect held when accounting for pet species (dogs, cats, other species) and number of pets owned. Compared to owners of other pets, dog owners reported higher well-being”	14
Antonacopoulos (51)	Canada	DO = 31; NDO = 35	Adults	Dog	“Examining the loneliness levels of adults in the general population who acquired a dog and a control group of non-dog guardians over an 8-month period using both an indirect and a direct measure of loneliness”	UCLA LS	Baseline 44.68 (13.25) 8 months 41.81 (12.10) Baseline 1.06 (1.21) 8 months 0.61 (0.80)	Baseline 46.86 (12.17) 8 months 46.91 (12.71) Baseline 1.00 (0.97) 8 months 1.23 (1.06)	“Changes in loneliness differed for owners and non-owners when assessed with a direct measure (1-item scale). Owners who adopted the dog had lower levels of loneliness from baseline to 8 months compared to non-owners. Loneliness when assessed by indirect measure, having a dog had no effect on loneliness (UCLA scale)”	22*
Ballin (74)	Sweden	DO = 199; NDO = 1,207	Older adults	Dog	“Investigated the associations of doing with accelerometer-measured in a population-based sample of 70-year-old women and men.”	GDS15	1.4 (1.7)	1.2 (1.8)	“DO was associated with higher levels of daily LPA, MVPA, and steps compared to non-owners.	15
Black (25)	USA	PO = 246; NPO = 47	Children	Any type of pet	“To investigate whether pet ownership and pet attachment are related to self-reported loneliness and social support among adolescents”	UCLA	33.7 (8.8)	39.5 (9.2)	“Pet owning adolescents had significantly lower loneliness scores and there was an inverse relationship between the level of bond with pet and levels of loneliness”	13
Bennett (24)	Australia	PO = 41; NPO = 27	Older adults	Dog; Cat; Large mamal; Bird; Fish	“To investigate whether the presence of a pet was associated with the presence and indicators of psychological well-being”	PWI-A PSS UCLA DASS-21: Depression Anxiety Stress	83.78 (13.1) 83.56 (8.2) 34.37 (9.5) 4.20 (5.0) 3.00 (3.7) 8.78 (7.4)	82.59 (11.3) 82.20 (7.1) 34.65 (6.9) 2.07 (2.5) 3.33 (3.1) 8.30 (6.5)	“Having a pet may not be associated with substantial differences in indicators of well-being in older people”	15
Bradley and Bennett (52)	Australia	PO = 114; NPO = 31	Adults who self-identified as having a chronic pain disorder	Dog; Cat	“Understand why therapy animals relieve pain in healthcare settings, but pet owners report greater discomfort and use more painkillers than people who do not own one or more pets”	DASS-21: Depression Anxiety Stress NPRS	18.10 (12.52) 11.28 (8.15) 17.47 (10.06) 6.14 (1.69)	11.74 (9.57) 10.39 (7.75) 15.35 (8.80) 5.92 (1.8)	“There was no significant difference between reported pain, anxiety, or stress levels in owners versus non-owners. Pet owners reported more depressive symptoms than non-owners, but owners with animals perceived as more friendly reported fewer depressive symptoms”	14
Branson (53)	USA	POcat = 41; NPO = 55	Older adults	Cat	“Determine if attachment to cats was associated with psychosocial responses (stress, depression, and loneliness)”	PStressS UCLA GDS MOCA	^b^*p* = 0.45 *p* = 0.83 *p* = 0.22 *p* = 0.37		“There were no significant changes between biopsychosocial and cognitive health outcomes with cat ownership”	14
Brkljacic (54)	Croatia	PO = 658; NPO = 3,883	Adults	Any type of pet	“Provide deeper insight into the relationship between pet-related life events and the subjective wellbeing of pet owners”	GLS BES SH	7.04 (1.91) 6.92 (2.10) 4.11 (0.79)	7.03 (2.02) 6.91 (2.09) 4.14 (0.82)	“There were no differences significant in subjective well-being indices, general life satisfaction and overall happiness, between the groups”	15
Canady and Sansone (55)	USA	PO = 153; NPO = 51	Adults	Any type of pet	“Examine whether companion animal owners report that having a companion animal would influence an important health decision, and whether existing social support and quality of the relationship with the companion animal might impact the likelihood of this occurring”	ISEL-12	35.4 (7.1)	34.5 (8.3)	“Having a pet can influence the decision to be hospitalized. It seems likely that social support acts as a buffer. Individuals with good social support entrust the care of their pets to others to receive the medical care they need”	14
Carr (75)	Canada	PO = 20; NPO = 36	Adults with chronic low back pain.	Any type of pet	“Evaluate the feasibility of surveying people with chronic low back pain to empirically assess the relationship between dog ownership and well-being for people with chronic low back pain”	NPRS ODI: Intensity ODI: Walking SF4 Depression Loneliness Emotional support Companionship	6.40 (1.67) 3.65 (0.93) 2.65 (0.93) 2.14 (0.79) 2.81 (1.38) 3.64 (0.98) 3.62 (1.15)	7.00 (1.45) 3.74 (0.95) 2.94 (0.92) 2.73 (1.10) 3.32 (1.51) 3.24 (1.26) 2.99 (1.27)	“Dog owners reported fewer depression and anxiety symptoms, and more social ties than non-dog owners. Living with a dog may be associated with improved well-being for people with chronic pain”	13
Cloutier and Peetz (56)	Canada	PO = 54; NPO = 62	Adults	Dog; Cat; Fish	“Compared pet owners and non-pet owners perceived relational quality, by assessing a variety of relationship quality facets, and examined whether there is any evidence of an association between pet ownership and quality of relationships”	QRS Responsiveness to Partner DAS RIMS	6.41 (0.56) 5.16 (0.52) 4.84 (0.55) 6.38 (0.76)	6.06 (0.74) 4.86 (0.61) 4.41 (0.73) 6.06 (0.71)	“Pet ownership was associated with several relationship benefits (higher overall relationship quality, partner responsiveness, adjustment, and relational investment) compared to couples without pets”	14
Curl et al. (57)	USA	DO = 188; NDO = 288	Older adults	Dog	“Examine the relationship between dog ownership, dog walking, and the emotional bond with a dog to contact with neighbors and life satisfaction in a nationally representative sample of adults in the United States over the age of 50”	SE LS SRH	1.86 (2.24) 2.78 (0.85) 2.26 (1.07)	1.79 (2.37) 2.91 (0.86) 2.26 (1.04)	“Dog ownership did not have a direct or indirect relationship on life satisfaction. However, time spent in dog walking was associated with the frequency of social interactions, which itself had a positive association with life satisfaction”	15
Endo (58)	Japan	DO = 254; POcat = 109; NPO = 2,230	Children	Dog; Cat	“Examine the effect of dog and cat ownership on the longitudinal trajectory of the mental well-being of adolescents”	WHO-5 (Dog) WHO-5 (Cat)	at age 10 79.42 (16.83) at age 12 77.53 (17.60) at age 10 80.04 (15.65) at age 12 69.69 (21.06)	at age 10 78.98 (16.63) at age 12 75.11 (18.87) at age 10 78.98 (16.63) at age 12 75.11 (18.87)	“Dog ownership and cat ownership differently predicted adolescent”s well-being. The well-being trajectory of dog owners was maintained through adolescence, while that of cat owners declined”	15
Feng (76)	United Kingdom	DO = 50; NDO = 497	Older adults	Dog	“To assess whether dog ownership in the older adult is associated with objectively measured physical activity”	HADS: Depression HADS Anxiety SF-36: Physical functioning SF-36: General health SF-36: Social functioning SF-36: Role Emotional SF-36: Mental health SF-36: Vitality SF-36: Pain SF-36: Role Physical	2.9 (2.6) 3.4 (2.8) 79 (17) 76 (17) 89 (24) 96 (12) 85 (13) 63 (22) 71 (28) 82 (27)	3.7 (2.7) 4.3 (3.2) 70 (23) 66 (21) 88 (22) 96 (11) 82 (13) 59 (20) 68 (26) 82 (25)	“The results suggest that dog ownership may motivate PA and enable older people to overcome many potential barriers to PA such as lack of social support. The effect of dog ownership on PA was independent of depression and perceived behavioral control but was mediated in part by general health and physical function”	16
Grajfoner (59)	Malaysia	PO = 202; NPO = 224	Adults	Dog; Cat	“Explore both the structure of companion animals in Malaysia and the effect of pets on mental health and wellbeing of Malaysians during the COVID-19”	WEMWBS DASS-21: Depression Anxiety Stress BRS CSE PANAS: Positive Negative	45.35 (10.58) 24.36 (9.66) 22.56 (8.79) 24.92 (9.34) 19.09 (3.19) 88.93 (16.00) 31.56 (7.63) 24.85 (7.95)	43.28 (9.81) 23.54 (9.50) 22.55 (8.91) 24.51 (9.23) 18.87 (3.17) 83.88 (18.74) 29.43 (7.16) 25.08 (6.98)	“Pet owners reported significantly better coping self-efficacy, significantly more positive emotions, and better psychological wellbeing”	14
Hajek and Konig (60)	Germany	DO = 63; POcat = 145; NPO = 952	Older adults	Dog; Cat	“Identify whether cat owners, dog owners, and individuals without pets differ in terms of depressive symptoms, loneliness, and social isolation among individuals in old age without a partner”	CES-D (dog) CES-D (cat) SI (dog) SI (cat) 11-DJGLS (dog) 11-DJGLS (cat) SRH (dog) SRH (cat) NPI (dog) NPI (cat) SF-36: physical health (dog) SF-36: physical health (cat)	7.3 (6.5) 7.8 (7.3) 1.6 (0.6) 1.7 (0.7) 1.7 (0.5) 1.8 (0.6) 2.7 (0.9) 2.6 (0.8) 3.5 (2.2) 3.5 (1.9) 74.3 (23.3) 75.7 (25.6)	7.2 (6.1) 7.2 (6.1) 1.7 (0.6) 1.7 (0.6) 1.8 (0.6) 1.8 (0.6) 2.6 (0.8) 2.6 (0.8) 3.4 (2.1) 3.4 (2.1) 72.0 (27.2) 72.0 (27.2)	“There was an association between owning a dog and social isolation (total sample) as well as loneliness (total sample and women)”	14
Hill (61)	Australia	PO = 392; NPO = 146	Adults	Any type of pet	“Explore the relationship between the HAB, perceived human social support, and resilience by assessing whether the HAB (human–animal bond) could moderate the impact of social support as a protective factor for resilience”	MSPSS CD-RISC	5.78 (0.96) 49.38 (11.85)	5.74 (0.98) 48.23 (11.48)	“There was no difference in levels of resilience between pet owners and non-owners, but social support was positively associated with resilience for both. The HAB was not a significant moderator between levels of social support and resilience for owners”	13
Kim and Chun (62)	Korea	PO = 8,708; NPO = 33,979	Adults	Dog; Cat	“Examine the association between companion animal ownership and overall life satisfaction, one measure of human well-being”	GLS	56.02 (10.25)	54.79 (10.68)	“Pet ownership was associated with higher levels of overall life satisfaction”	15
Mičková (77)	Czech Republic	DO = 26; NDO = 18	Older adults	Dog	“To see if dog ownership affects physical activity, sleep, and self-reported health in a group of older adult people”	SF-36: General health SF-36: Health change SF-36: Physical functioning SF-36: Social functioning SF-36: Emotional SF-36: Pain SF-36: Vitality SF-36: Role Emotional) SF-36: Role Physical	72 (15) 47 (11) 88 (12) 90 (18) 80 (12) 78 (19) 67 (15) 86 (29) 85 (27)	46 (14) 43 (14) 72 (22) 76 (18) 69 (13) 62 (22) 47 (6) 83 (26) 71 (33)	“A positive effect on their overall health assessed by SF-36 was observed in most of the monitored parameters. The results suggest that dog walking affects the overall PA of older adults and it brings positive effects on the quality of life”	14
Mueller (78)	USA	PO = 910; NPO = 310	Children	Any type of pet	“Contribute to the emerging research on companion animals and mental health during the pandemic by assessing the relationships between pet ownership, pet attachment, loneliness, and stress coping for adolescents”	LS	Time 1 1.43 (0.52) Time 2 1.62 (0.60)	Time 1 1.47 (0.53) Time 2 1.49 (0.52)	“The results of this study do not support the presence of a buffering effect of companion animals on loneliness for adolescents”	16
Muldoon (63)	United Kingdom	DO = 2,784; PO = 1992 NPO = 1887	Children	Any type of pet	“To see if within the broader population of children and adolescents, to what extent attachment to dogs: (a) is stronger than attachment to other pets; (b) differs from emotional connections to other animals; and (c) is associated with any specific welfare benefits”	SAPS (dog) SAPS (pet)	^b^d = 0.68 d = 0.25		“Pets, especially dogs, have an impact on well-being when a strong emotional bond is present”	14
Muraco (64)	USA	PO = 1,039; NPO = 1,326	Adults LGBT	Any type of pet	“Understanding whether having a pet is related to perceived social support and social network dimension”	PSS	3.19 (0.76)	2.99 (0.8)	“There is evidence that pets can increase feelings of social support for people with disabilities and limited social networks”	14
Phillipou (43)	Australia	PO = 138; NPO = 125	Adults	Dog; Cat	“Explore the mental health effects of pet ownership during the COVID-19 pandemic lockdown”	DASS-21: Depression Anxiety Stress UCLA BRS EUROHISQoL	13.15 (10.79) 6.38 (7.48) 13.64 (9.43) 9.1 (3.09) 3.2 (0.99) 27.2 (7.27)	11.57 (10.35) 6.14 (7.66) 13.15 (8.82) 8.53 (2.65) 3.3 (0.89) 28.81 (6.61)	“Contrary to expectations, the results suggest that during a specific situation such as a pandemic, pets may contribute to an increased burden on owners and contribute to a worse quality of life”	14
Pruchno (65)	USA	DO = 1,160; POcat = 947; POdog+ cat = 441; NPO = 2,954	Adults	Dog; Cat	“Examined the associations among human social relationships, owning a dog or a cat, and successful aging”	SS (Dog) SS (Cat) SS (Dog; cat) SSA (Dog) SSA (Cat) SSA (Dog; cat)	4.3 (0.8) 4.2 (0.9) 4.2 (0.9) 23.7 (4.3) 23.1 (4.7) 23.1 (4.4)	4.2 (0.8) 4.2 (0.8) 4.2 (0.8) 23.4 (4.4) 23.4 (4.4) 23.4 (4.4)	“Findings carry practical implications for supporting pet ownership of older people, suggesting that dogs have a positive association with successful aging”	14
Ramírez and Hernández (66)	USA	DO = 377; NDO = 225	Adults	Dog	“Compared the perceived health, perceived stress, life satisfaction, happiness and psychosomatic symptoms in two equivalent groups that differed only in dog ownership”	LSS SHS PHQ PStressS SF-12: Mental health SF-12: Physical health	16.0 (2.8) 22.7 (3.4) 5.3 (3.9) 18.0 (7.3) 51.0 (8.2) 52.8 (6.9)	15.6 (3.0) 22.5 (3.7) 6.0 (3.9) 20.0 (7.2) 48.7 (8.7) 51.9 (7.5)	“Dog owners perceived themselves as healthier but not happier than non-dog owners”	14
Reis (67)	Portugal	DO = 1764; POcat = 739; POdog+ cat = 901 PO = 520; NPO = 1,211	Children	Any type of pet	“Identify in a large national representative sample of Portuguese adolescents, the percentage of adolescents that have pets, what kind of feelings pets provide, differences by gender and age (through school grades) and to verify whether adolescent health, well-being, life satisfaction, and psychological symptoms are associated with having a pet (in particular dogs or cats)”	Kidscreen-10 (Dog) Kidscreen-10 (cat) Kidscreen-10 (Dog; cat) Kidscreen-10 (pets) LSS (Dog) LSS (Cat) LSS (Dog; cat) LSS (pets) PS (Dog) PS (Cat) PS (Dog; cat) PS (pets)	39.41 (6.62) 38.30 (6.98) 38.44 (6.64) 39.05 (6.70) 7.50 (1.96) 7.18 (1.99) 7.34 (1.94) 7.43 (1.95) 6.55 (1.41) 6.38 (1.46) 6.39 (1.45) 6.54 (1.41)	39.03 (6.79) 39.03 (6.79) 39.03 (6.79) 39.03 (6.79) 7.42 (1.93) 7.42 (1.93) 7.42 (1.93) 7.42 (1.93) 6.52 (1.42) 6.52 (1.42) 6.52 (1.42) 6.52 (1.42)	“Having a dog was associated with a higher socio-economic status. Better perception of well-being. More life satisfaction and less psychological symptoms”	15
Roux and Wright (68)	Africa	PO = 3,108; NPO = 221	Adults	Dog; Cat	“Investigate whether pet attachment was related to perceived stress and life satisfaction in a sample of South Africans”	PSS SWLS	17.9 (7.16) 23.4 (6.79)	18.1 (6.09) 22.9 (7.11)	“Dog owners were significantly more attached to their dogs. Significantly more satisfied with their lives and had significantly less stress than cat owners”	14
Taniguchi (13)	Japan	PO = 1,545; NPO = 6,377	Older adults	Dog; Cat	“Evaluated physical function. PA. social function. and psychological function of a population of community-dwelling older Japanese dog and cat owners”:	GDS-5 WHO-5	1.2 (1.3) 62.5 (23.3)	1.3 (1.3) 60.2 (24.4)	“Caring for a dog or cat might be an effective health promotion strategy to increase physical activity and facilitate social participation among older adults”	16
Teo and Thomas (70)	Australia	DO = 332; PO = 332; NPO = 176	Adults	Any type of pet	“Compare multi-faceted QOL. psychological distress. and psychopathology of pet owners and non-pet owners”	DASS-21 Depression(dog) Depression (pets) Anxiety (dog) Anxiety (pets) Stress (dog) Stress (pets) BSI (dog) BSI (pets) WHOQOL (dog) WHOQOL (pets)	8.67 (8.87) 7.85 (9.50) 6.01 (6.86) 5.37 (7.21) 11.08 (8.29) 8.65 (7.80) 0.79 (0.62) 0.67 (0.59) 58.30 (9.04) 58.44 (8.71)	7.05 (8.36) 7.05 (8.36) 5.56 (6.12) 5.56 (6.12) 8.78 (7.57) 8.78 (7.57) 0.66 (0.58) 0.66 (0.58) 58.54 (9.82) 58.54 (9.82)	“Pet owners and non-owners did not differ significantly in terms of well-being”	14
Watson and Weinstein (71)	USA	PO = 42; NPO = 42	Adults women	Dog; Cat	“Explore the potential psychological benefits of pet ownership among working women”	BDI STAI STAXI: state STAXI: trait	5.5 (4.1) 32.5 (10.2) 10.9 (2.5) 17.5 (4.6)	6.0 (4.5) 35.9 (9.4) 11.0 (1.7) 18.8 (4.2)	“Results revealed that there were no significant differences between owners” and non-owners”	13
Wright (72)	United Kingdom	DO = 14; NDO = 26	Children with autism	Dog	“Investigate the potential of dogs to improve family functioning in families with children with autism and explore the effects of pet dogs on anxiety in these children”	CAS	Baseline 0.33 (0.05) Follow-Up 0.30 (0.04)	Baseline 0.26 (0.03) Follow-Up 0.23 (0.03)	“Acquisition of a dog may be associated with improvements in family functioning and child anxiety”	23*
Wright (73)	USA	DO = 36; POcat = 15; NDO = 47; NPO = 9	Adults LGBT	Dog; Cat	“Understanding the relationship between pet companionship and quality of life outcomes in sexual minority prostate cancer survivors”	SF-12	46.05 (10.34) 50.8 (11.4)	48.5 (10.26) 51.4 (8.34)	“Pet companionship may be a net stressor for gay and bisexual men following prostate cancer treatment”	14

PO, pet owner; NPO, Non-pet owner; N, sample size; SD, standard deviation; M, mean. instruments: BDI, beck depression inventory; BES, subjective happiness scale; CAS, children’s anxiety scale; CD-RISC, the Connor-Davidson resilience scale; CES-D, depression scale; DAS, dyadic adjustment scale; DASS-21, depression anxiety stress scale; GDS-5, geriatric depression scale; GHQ-30, 30-item general health questionnaire; GLS, general life satisfaction; HADS, hospital anxiety and depression score; ISEL-12, interpersonal support evaluation; LS, loneliness scale; LSS, life satisfaction scale; MOCA, the Montreal cognitive assessment; MSPSS, multidimensional scale of perceived social support; NPI, number of physical illnesses; NPRS, numeric pain rating scale; ODI, Oswestry disability index; PHQ, patient health questionnaire; PLF, presence of life meaning; PS, psychological symptoms; PSS, perceived social support; PStressS, perceived stress scale; PWI-A, personal wellbeing index-adult; QRS, quality of relationship scale; RIMS, Rusbult investment model scale; SAPS, short attachment to pets scale; SE, social engagement; SF4, anxiety and depression scale (PROMIS); SF-12, short form health survey; SF-36, health survey; SH, subjective health; SHS, subjective happiness scale; SI, social isolation; SRH, self-rated health; SS, social support; SSA, subjective successful aging; STAI, StateTrait anxiety inventory; STAXI, state–trait anger expression inventory; SWLS, satisfaction with life scale; UCLA, loneliness scale; WHO-5, Weel-being index; 11-JGLS, 11-item De Jong Gierveld- Loneliness scale; PSS, perceived stress scale.

* Down’s and Black tool uses 27 criteria.

a Articles arranged in alphabetical order.

b General results selected due to the absence of mean and standard deviation measures.

### 3.3. Pet influence on physical activity

**Figure 2:**
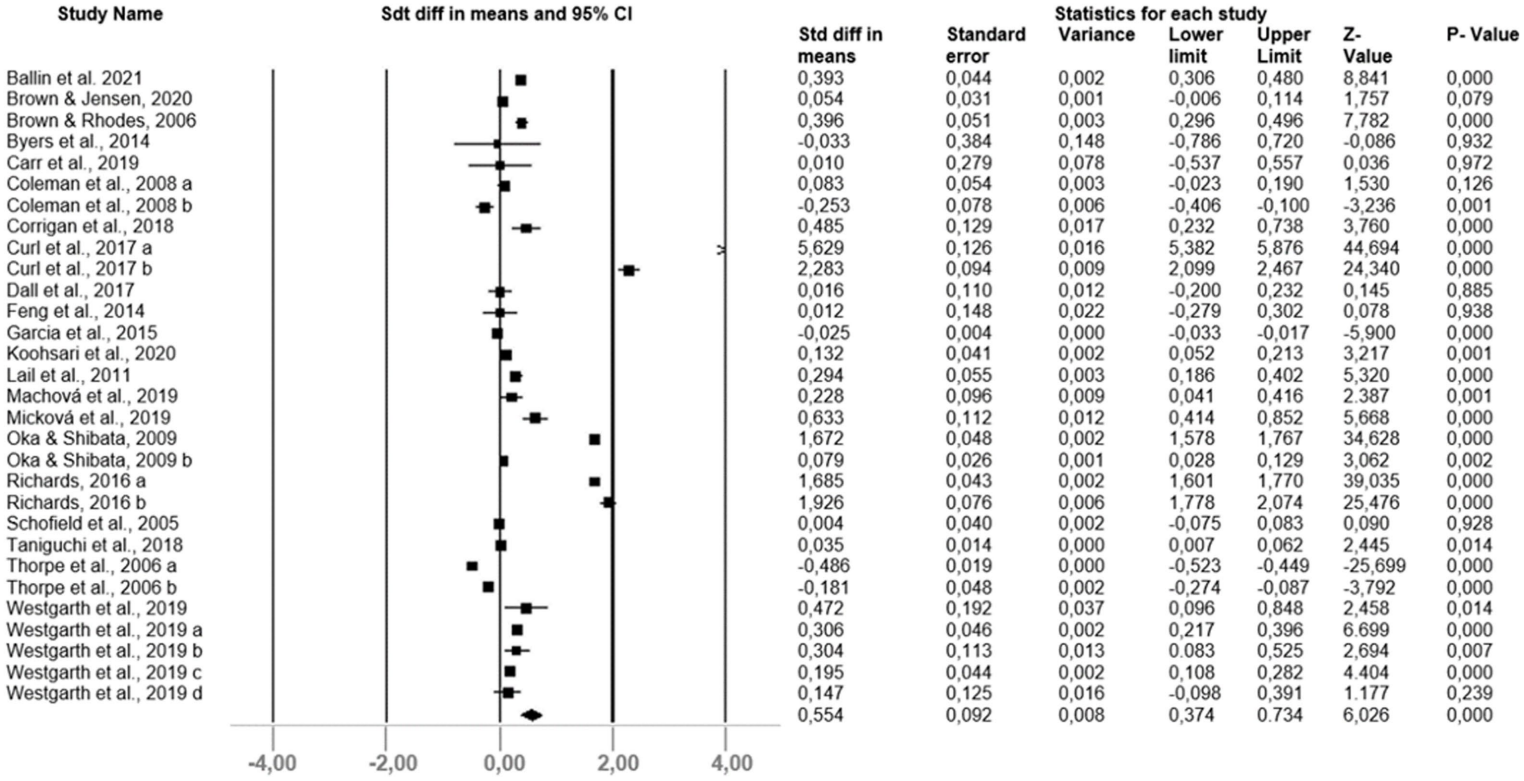
Forest plot of pet influence in physical activity ownership.

The main analysis showed that pets had a significant and positive effect on the PA of owners compared to non-owners, with an effect of moderate and significant magnitude (Cohen’s d = 0.554; *p* = 0.000; Figure 2). The studies showed high heterogeneity (I^2^ = 99.586%; *p* = 0.000). Although the asymmetry in the funnel plot indicated a likelihood of publication bias, it was not confirmed by Begg’s (*p* = 0.06171) and Egger’s (0.21448) tests.

Regarding the analysis of moderating variables by owner’s age group there was a significant and positive influence but of low magnitude in adults (Cohen’s d = 0.009; 95% CI [0.001–0.016]; *p* = 0.000), and older adults (Cohen’s d = 0.009; 95% CI [0.135–0.184]; *p* = 0.000). No significant values were observed in children. The results indicated a moderate and high heterogeneity (I^2^ = 99.932%, I^2^ = 99.063%, respectively, for adults and older adults).

Considering the analysis of the PA moderator variables, the frequency of performing physical activity showed a high magnitude (Cohen’s d = 1.386; 95% CI [1.297–1.476]; *p* = 0.000), with high heterogeneity (I^2^ = 99.574%). The moderator variables of PA counts (Cohen’s d = 0.423; 95% CI [0.295–0.551]; p = 0.000) and Met (Cohen’s d = 0.147; 95% CI [0.124–0.171]; p = 0.000) showed low but significant effect magnitude. Duration did not show a significant value. The heterogeneity presented in the significant variables was high (I^2^ = 99.917%, I^2^ = 72.678%, respectively for met and counts).

The analysis of the moderating variables regarding the instruments used for measuring PA revealed a small effect magnitude for the objective (Cohen’s d = 0.180; 95% [0.136–0.224)]; *p* = 0.000) and subjective measure (Cohen’s d = 0.018; 95% [0.010–0.025]; p = 0.000), but significant. The heterogeneity presented was high for both objective (I^2^ = 81.523%) and subjective (I^2^ = 99.923%) moderating variables.

### 3.4. Pet influence on mental health

**Figure 3:**
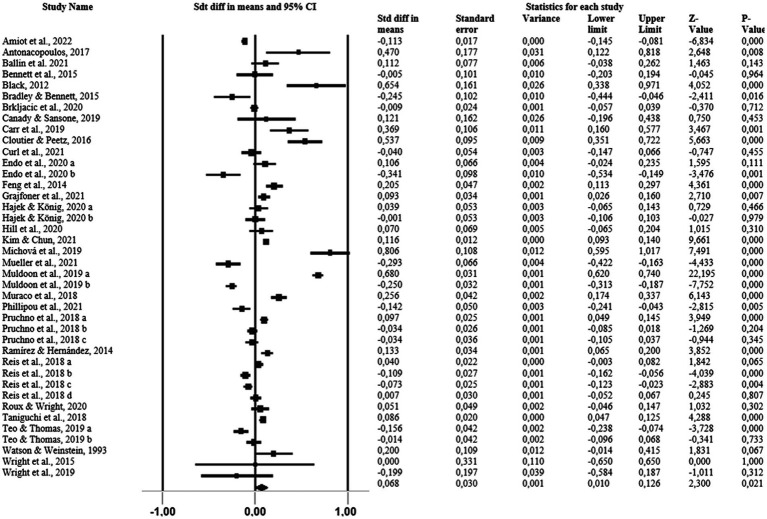
Forest plot of pet influence on mental health.

In the main analyses, it was found that pets have a significant and positive effect on owners. Additionally, a significant and positive effect on owners’ mental health was reported, albeit of low magnitude (*p* = 0.021; Cohen’s d = 0.068; Figure 3). The studies showed high heterogeneity (I^2^ = 95.987%; *p* = 0.000). However, the symmetric funnel plot analysis revealed a low risk of publication bias, as evidenced by Begg’s (*p* = 0.11060) and Egger’s tests (*p* = 0.34245) (Supplementary material B).

Owner’s age group as a moderating variable has a significant and positive influence but of low magnitude in children (Cohen’s d = 0.030; 95% CI [0.009–0.05; *p* = 0.005]), adults (Cohen’s d = 0.037; 95% CI [0.0024–0.05]; *p* = 0.000), and older adults (Cohen’s d = 0.091; 95% CI [0.061–0.121]; p = 0.000). The results indicated a moderate and high heterogeneity of moderating variables by owner’s age group (I^2^ = 98.397%, I^2^ = 77.605%, I^2^ = 60.934%, respectively for children, adults, and older adults).

Considering the analysis of moderating variables by mental health, all showed a low effect magnitude but with a significant and positive influence on the humor and self-regulation (affections, emotions, relationships; Cohen’s d = 0.241; 95% CI [0.203–0.280]; *p* = 0.000), social support (Cohen’s d = 0.100; 95% CI [0.064–0.137]; p = 0.000) life satisfaction and happiness (Cohen’s d = 0.063; 95% CI [0.044–0.081]; p = 0.000). Loneliness had a significant influence, but a negative effect (Cohen’s d = −0.036; 95% CI [−0.088–0.017]; *p* = 0.005), anxiety, loneliness depression, stress, life satisfaction and happiness, social support, quality of life, health and well-being, general mental health and resilience showed low magnitude and non-significant values. Despite exploration among the moderating variables, heterogeneity remained moderate to high (I^2^ = 0.000%, I^2^ = 82.205%, I^2^ = 65.479%, I^2^ = 66.963%, I^2^ = 83.883%, I^2^ = 67.735%, I^2^ = 740,739%, I^2^ = 76.147%, I^2^ = 97.371%, respectively for anxiety, loneliness depression, stress, life satisfaction and happiness, social support, quality of life, health and well-being, general mental health and resilience, humor and self-regulation (affections, emotions, relationships).

## 4. Discussion

To our knowledge, this is the first comprehensive meta-analysis to investigate the influence of pet ownership on owners’ daily PA levels and mental health. The main findings indicate a moderate positive impact of pets on PA compared to non-pet owners (NPO).

Among the PA moderating variables, frequency showed a highly significant effect, indicating that pet owners have a higher frequency of physical activity than NPO. However, no significant impact of pet ownership on mental health compared to NPO was found. One of the most promising results of this meta-analysis is the evidence that pet owners are more physically active than NPO, which may be related to pet care such as walking and going outside. A previous study (19) found that dog owners walk moderately more than non-dog owners. Of all the analyzed studies, five were conducted in dogs and other different species (13, 29, 77, 87, 90). The analysis of these studies showed that the benefits related to PA were more evident in dog owners than in owners of other pets. These findings led us to analyze PA specifically regarding dog owners DO and NDO.

About the moderating variables of PA (frequency, duration, counts, and mets), PO significantly had a higher frequency of walking. However, duration was not significant, as found in a previous meta-analysis (19). Owners with a stronger attachments to their dogs were more likely to walk with them, but at a shorter distance than owners with weaker pet attachments (17). These results may be based on the owner’s commitment to meet the pet’s needs, which may lead to an increase in the owner’s willingness and frequency to take a walk, even if it is not perceived, as opposed to non-owners. On the other hand, it is possible that dog characteristics, such as age and breed, could influence the relationship between physical activity and mental health outcomes (92). For example, younger dogs may require more physical activity than older dogs, which could demand more attention and owners’ general activity. Moreover, it is known that some breeds have higher exercise requirements than others (92). Future studies could take into account factors such as age, breed, and dogs’ physical activity needs, and how they influence health outcomes of pet owners.

Objective and subjective measurement methods revealed a significant, but low effect size. For the assessment of owners’ PA, most previous studies have used subjective physical activity measures, with only eight studies using objective assessment measures (10, 74, 76, 77, 80, 81, 84, 89). One of the limitations of subjective measures is that they are based on the perception or recall of PA performed before the date of completing the questionnaire, which may generate bias in the results or overestimation compared to objective measures (93). Comparative studies comparing the two measurement methods have found that the results obtained through the IPAQ are not reflected in the PA measurements with the accelerometer (77, 79). Despite our results covering both methodologies, subjective measures were the most commonly reported method among studies, similar to the meta-analysis conducted in 2013 (19). Although studies conducted with objective measures have been increasing, more studies that examine the application of these methods are needed, particularly studies that quantify both pet and owner levels of PA. Moreover, such an analysis would allow us to establish a more reliable role of pets in owners’ PA and possibly define guidelines for the population that can benefit the most from owning a pet.

Besides the relationship between pet ownership and physical health, there may also be a relationship with mental health. There is some evidence that suggest that physical activity and pet ownership can both have positive effects on mental health outcomes (11). Engaging in regular physical activity has been shown to be associated with improved mental health, including decreased symptoms of depression and anxiety, as well as increased feelings of well-being and self-esteem (32). Thus, it is possible that physically active pet owners may have better mental health outcomes compared to non-pet owners, as they may benefit from both the physical activity and the social support and companionship provided by their pets (28, 94). However, more research is needed to fully understand the complex relationship between physical activity, pet ownership, and mental health outcomes.

Regarding the relationship between mental health and pet ownership, this meta-analysis included 135 results from 32 studies, whose methodological quality ranged from good to excellent. Despite the significant influence of pets on owners’ mental health, it must be considered that the effect size was low. The high heterogeneity of the sample characteristics, the variables used to measure mental health, and other methodological issues might explain this low effect size. Nevertheless, this result has already been described in previous systematic reviews (91, 95).

To better understand the heterogeneity found, various moderating variables related to mental health were considered (95). Among these moderating variables, only loneliness, social support, life satisfaction, happiness, and mood and self-regulation were significantly related to pet ownership.

Regarding loneliness and social support, this meta-analysis suggests that PO are more likely to experience loneliness than NPO, but owning a pet can confer greater social support to the owner. Despite the differences between these concepts, they complement each other. Loneliness refers to the discrepancy between actual and desired social relationships. Social isolation (included in the social support moderating variable of this meta-analysis) arises in the absence of such contact with society (96). Kretzler (3) suggests that a pet tends to be associated with an increased frequency of social interactions, allowing for the increase of social and community ties (57), which may favor increased feelings of belonging and decreased loneliness and social isolation (70). Among the activities shared between pets and owners, walking and visits to parks appear to be most strongly associated with the social support felt by owners (13). Dogs are the most frequently reported pets in these activities (13, 57, 75) and seem to produce better results compared to other pets (65, 96). However, these differences between species are mostly dependent on the type of activities performed with the pet. Therefore, it may be important to consider other types of daily life activities with pets in future studies, as they may also promote greater social support for owners and decrease feelings of loneliness.

The literature suggests that pets may play a beneficial role in providing social support and companionship, particularly for older adult individuals who live alone (24, 51). However, while pets may serve as a form of social support, they cannot completely alleviate feelings of loneliness. Despite these findings, the low effect size of the relationship between pet ownership and mental health can be attributed to the high heterogeneity of the samples. Studies with individuals with chronic low back pain (75), members of the LGBT community (64), and those experiencing the COVID-19 pandemic (43, 78) have further demonstrated the high variability of samples. Certain contexts may even contribute to increased inconsistencies and incoherence in the role of pets in social isolation and feelings of loneliness. For instance, during the COVID-19 pandemic, social isolation was not significantly associated with pet ownership, but loneliness may have been reduced (3). Further research is needed to fully understand the impact of pets on social support and levels of loneliness.

This meta-analysis also indicates that pets may promote greater life satisfaction and happiness in their owners. The concept of life satisfaction is subjective and may depend on individuals’ experiences. Curl (57) reported that pet owners experience greater social engagement and life satisfaction, especially in the older adult population. Additionally, pet owners who experienced the death of a pet during the previous year were significantly less happy and satisfied compared to those who did not have a pet and those who did not experience the death of a pet (54). On the other hand, it is also plausible to consider that owners’ personalities, conditions of the pets’ presence, as well as expenses associated with the pet’s care, might influence owners’ life satisfaction. Therefore, it is important to understand the relationship between pet ownership and life satisfaction and happiness, considering different variables such as life satisfaction before and after the adoption of the pet.

This meta-analysis also shows that the presence of a pet may lead to better mood, coping skills, affection, and relationships, particularly regarding humor and emotions. Moreover, having a pet throughout life was predictive of more positive relationships (56). However, high heterogeneity was found, which could be explained by the attachment to the pet, as the human-animal bond may differ among members of the same family, influencing their responses to the same questionnaire. Most analyzed studies did not control for this variable, which makes it difficult to understand the possible influence of the human-animal bond. Therefore, in future studies, it will be important to consider this variable.

Concerning the remaining moderator variables, no significant effect of pets was found, and the magnitude of the effect was low. These results may be due, in part, to the diversity of instruments and methodological procedures used in the included studies. In fact, in the different studies analyzed the variable mental health and resilience were quantified by using different scales, which most frequently was the Short Form Health Survey with 36 items (43, 59, 61), with Whight (73) using a reduced version with 12 items, the Moca (53), Patient Health Questionnaire (66), Psychological Symptoms (67), and BSI- Brief Symptom Inventor (70) were also used, along with 3 other studies (43, 59, 61) that assessed resilience. This fact may cause bias and variability, as it depends on the reading and interpretation of the self-administered questionnaire. Therefore, the high variety of instruments used to measure the same or different mental health variables was probably the main reason for the high heterogeneity observed. Similar results were observed for the depression and anxiety variables.

Therefore, 12 studies were included in this meta-analysis that explored the effect of pet presence on depressive symptoms (13, 24, 43, 52, 53, 59, 60, 70, 71, 74–76), and 8 studies on anxiety (24, 43, 52, 59, 70–72, 76). However, no significant effect was observed, which is consistent with other reported literature (91, 95). Symptoms of anxiety and depression are frequently analyzed together since they are highly comorbid and share common etiological processes (97). In this meta-analysis, only one study (72) analyzed anxiety independently of depression. In the remaining studies, the authors used the same instrument to analyze both variables: the Depression, Anxiety, and Stress scale (24, 43, 52, 59, 70) and the Hospital Anxiety and Depression Scale (76).

Regarding the influence of pets on the owner’s quality of life, health, and well-being, no significant results were found, contradicting a previous systematic review that reported the potential benefits of pets to impact owner well-being (28). Once again, different scales and procedures were used in the reviewed studies. Quality of life was measured in 17 studies using subjective health items (57, 96), the European Health Interview Surveys-Quality of Life questionnaire (43), the Warwick-Edinburgh Mental Well-Being Scale (59), the Kidscreen-10 index (67), items from the SF-36 scale (vitality, pain, and role physical) (30, 76, 77), and items from the shortened version SF-12 (physical health) (66, 73). The physical health scales were used in studies on physical illnesses (52, 65, 75, 96). The World Health Organization Quality of Life Instrument (70) and the Well-being Index (58) was also used to measure well-being.

Quality of life, health, and well-being questionnaires may be subject to bias due to the subjectivity of interpretation inherent in the different dimensions evaluated. Moreover, the confounding factors considered by the authors, depending on the study goal, are different in each study, which could also be a limitation of our analysis.

Overall, although this meta-analysis did not aim to understand the effect of different pet species on mental health, it is worth mentioning that they seem to have an influence. Pruchno (65) found a higher positive association between quality-of-life outcomes and dog ownership than cat ownership, while Hajek (96) found a similar association regarding social isolation and loneliness. In another study with Portuguese adolescents, pets were associated with a better perception of well-being, more life satisfaction, and overall mental health. However, when analyzed by species, dogs showed more evident results (67). This may also contribute to explaining the high heterogeneity of the obtained results.

## 5. Limitations

This meta-analysis has identified several limitations and methodological issues that limit the generalizability of the results. These flaws include the absence of randomized controlled trials and a small number of longitudinal studies. Additionally, there is a lack of studies that compare health-related variables before and after pet ownership. It is also important to differentiate between participants, distinguish between the main and secondary responsible owners, and to sure attachment to the pet. Furthermore, it would be valuable to include other moderating variables such as age group, gender, economic factors, social status, ethnicity, and pet species to reduce the heterogeneity of the analysis. Finally, the use of diverse instruments to assess mental health and physical activity increases the heterogeneity of the results.

## 6. Conclusion

In general, pet ownership has been found to have a positive influence on owners’ physical activity, with pet owners showing a higher frequency of physical activity than non-owners. However, pets do not seem to have a significant impact on owners’ mental health. There were some moderating variables related to mental health, such as loneliness, social support, life satisfaction, happiness, mood, and self-regulation, which were significantly associated with pet ownership but with low effect sizes. This suggests that pet owners may have higher levels of social support, life satisfaction, happiness, mood, and self-regulation and lower levels of loneliness than non-owners.

The results of this meta-analysis provide a nuanced understanding of the potential impact of pets on owners’ mental health and physical activity from a one health perspective.

We suggest that future researchers explore theoretical frameworks and methodological approaches that can explain the uniqueness of the relationships between pets and people, and how these influence them.

## Author contributions

CM, JS, MM, MP, and LC: the conception of the research, the design of the research protocol, and review of the final draft of the manuscript. CM, JS, and MM: literature review and manuscript drafting. CM and MM: publication search. CM, JS, MM, and AC: publication screening and data extraction. LC and LS: third and fourth reviews. AC: statistical analysis. CM, AC, JS, and LS: data analysis and interpretation of results. All authors contributed to the article and approved the submitted version.

## Funding

This work was funded by the R&D&I project “oneHcancer – One health approach in animal cancer,” operation no.: NORTE-01-0145-FEDER-000078, co-funded by the European Regional Development Fund (ERDF) through NORTE 2020 (North Portugal Regional Operational Program 2014/2020).

## Conflict of interest

The authors declare that the research was conducted in the absence of any commercial or financial relationships that could be construed as a potential conflict of interest.

## Publisher’s note

All claims expressed in this article are solely those of the authors and do not necessarily represent those of their affiliated organizations, or those of the publisher, the editors and the reviewers. Any product that may be evaluated in this article, or claim that may be made by its manufacturer, is not guaranteed or endorsed by the publisher.

## Supplementary material

The Supplementary material for this article can be found online at: https://www.frontiersin.org/articles/10.3389/fpubh.2023.1196199/full#supplementary-material
