# Supplementary material for: Pet’s influence on humans’ daily physical activity and mental health: a meta-analysis
Source: Front Public Health. 2023 May 30;11:1196199. doi: 10.3389/fpubh.2023.1196199 (PMC10262044; doi:10.3389/fpubh.2023.1196199)
Supplement: Supplementary file 2 [file Data_Sheet_2.docx]

Supplementary material B- Funnel plots and subgroup analyses


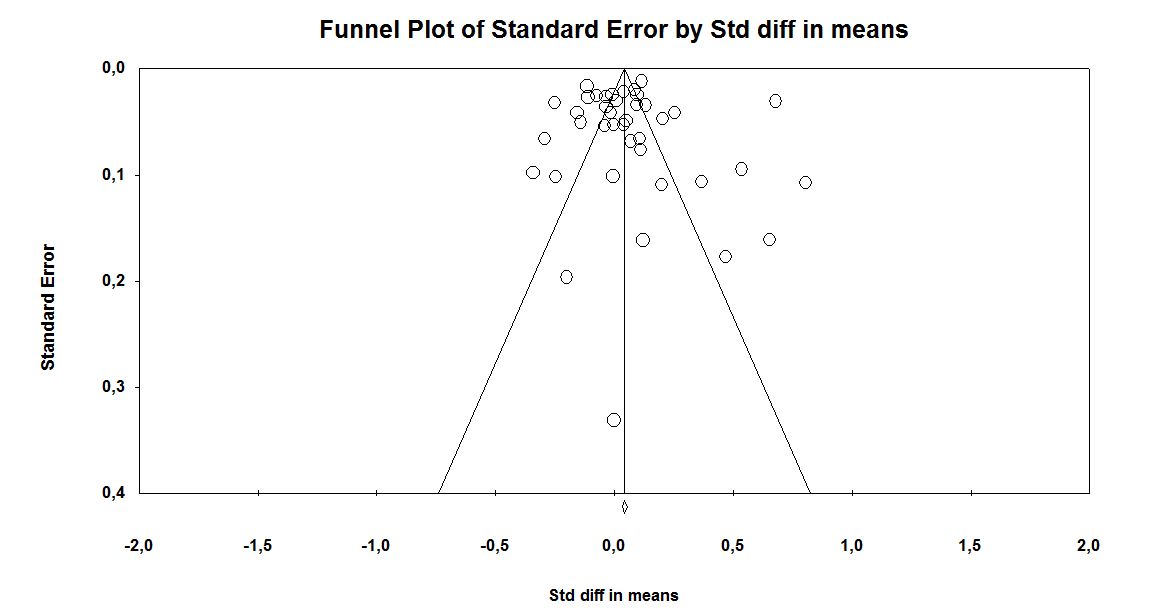


**A**

**B**


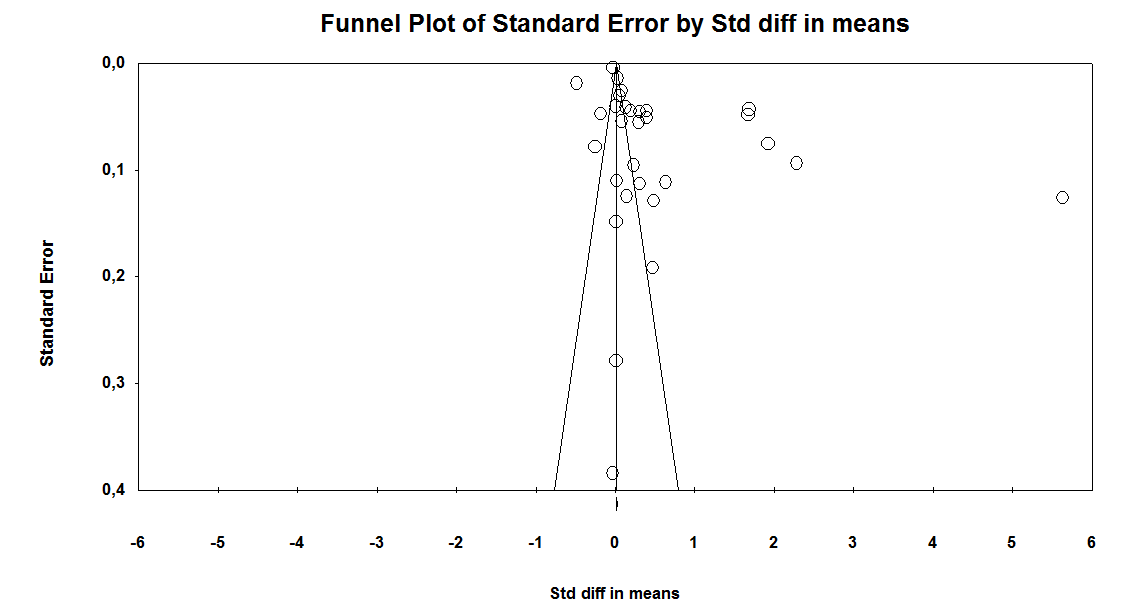


Figure B 1- Funnel plot A- mental health and funnel plot B – physical activity.

Table B.1- Subgroup analyses on mental health.

| Subgroup analysis | | No. of results | 𝑑 of cohen; (95% CI) | *p*-value | I^2^ |
| --- | --- | --- | --- | --- | --- |
| Age of sample | Children | 19 | 0.030; (0.009-0.051) | 0.005 | 98.397 |
|  | Adults | 75 | 0.037; (0.0024 – 0.51) | 0.000 | 77.605 |
|  | Old adults | 43 | 0.091; (0.061-0.121) | 0.000 | 60.934 |
|  |  | | | | |
| Mental health | Anxiety | 9 | 0.023; (-0.063 – 0.109) | 0.596 | 0.000 |
|  | Loneliness | 10 | -0.083; (-0,142 – -0.025) | 0.005 | 82.205 |
|  | Depression | 13 | 0.029; (-0.014 – 0.073) | 0.183 | 65.479 |
|  | Stress | 9 | -0.036; (-0.088 – 0.017) | 0.182 | 66.963 |
|  | Life satisfaction and happiness | 13 | 0.063; (0.044 – 0.081) | 0.000 | 83.883 |
|  | Social Support | 12 | 0.100; (0.064 – 0.137) | 0.000 | 67.735 |
|  | Quality of life, health and well-being | 39 | -0.007; (-0.029 – 0.014) | 0.489 | 74.739 |
|  | General mental health and resilience | 17 | -0.014; (-0.047 – 0.018) | 0.385 | 76.147 |
|  | Humor and self-regulation (affections, emotions, relationships) | 13 | 0.241; (0.203 – 0.280) | 0.000 | 97.371 |

Table B. 2- Subgroup analyses for studies on physical activity.

| Subgroup analysis | | No. of results | Cohen’s *d*; (95% CI) | p-value | I^2^ |
| --- | --- | --- | --- | --- | --- |
| Age of sample | Children | 5 | 0.333 (0.015 – 0.650) | 0.040 | 45,263 |
|  | Adults | 65 | 0.009 (0.001 – 0.016) | 0.027 | 99.932 |
|  | Old adults | 27 | 0.159 (0.135 – 0.184) | 0.000 | 99.063 |
|  |  | | | | |
| Physical Exercise | Counts | 5 | 0.423 (0.295 – 0.551) | 0.000 | 72,678 |
|  | Frequency | 9 | 1.386 (1,297- 1.476) | 0.000 | 99,574 |
|  | Met | 12 | 0.147 (0.124 – 0.171) | 0.000 | 99.917 |
|  | Duration | 71 | -0.003 (-0.011 – 0.005) | 0.455 | 99.914 |
|  |  |  |  |  |  |
| Instrument | Objective Measure | 21 | 0.180 (0.136 – 0.224) | 0.000 | 81,523 |
|  | Subjective Measure | 76 | 0.018 (0.010 – 0.025) | 0.000 | 99.923 |
